# Acute calcific periarthritis of the proximal phalangeal joint on the fifth finger: A case report and literature review

**DOI:** 10.1097/MD.0000000000021477

**Published:** 2020-07-31

**Authors:** Yuji Tomori, Mitsuhiko Nanno, Shinro Takai

**Affiliations:** a Departments of Orthopaedic Surgery, Nippon Medical School Musashi Kosugi Hospital, Kanagawa; b Departments of Orthopaedic Surgery, Ukima Central Hospital; c Departments of Orthopaedic Surgery, Nippon Medical School Hospital, Tokyo, Japan.

**Keywords:** acute calcific periarthritis, benign calcifying lesion, case report, phalanges, proximal phalangeal joint, pseudotumor

## Abstract

**Rationale:**

Acute calcium deposits, including acute calcific periarthritis or acute calcific peritendinitis, are benign calcifying soft tissue lesions that have a self-resolving course. These calcifying lesions usually develop in the shoulder, while acute calcific periarthritis in the digits is uncommon. When acute calcific periarthritis involves the digits, the lesion occasionally mimics other benign calcifying or ossifying lesions and can easily be misdiagnosed, resulting in unnecessary diagnostic studies and treatment. We present a rare case of acute calcific periarthritis around the proximal phalangeal joint of the left fifth finger that took a long time to spontaneously resolve, and review previous reports of similar cases.

**Patient concerns:**

A 69-year-old woman complained of longstanding pain and swelling of the fifth finger of the left hand. She had visited several clinics and hospitals and had been treated with analgesics and splinting for more than 2 months, but the pain in the finger had gradually worsened.

**Diagnoses:**

Blood chemistry analysis showed no signs of inflammation or other abnormalities. Radiographs revealed a well-defined subcutaneous calcifying lesion without bony destruction, suggesting a benign calcification process. Computed tomography and magnetic resonance imaging led to a diagnosis of acute calcific periarthritis of the proximal interphalangeal joint of the fifth finger.

**Interventions:**

An excisional biopsy was recommended to achieve a definitive diagnosis, but this was declined by the patient. Thus, no invasive treatments were administered, and she was treated with analgesics and encouraged to massage the affected finger.

**Outcomes:**

The pain gradually improved, and follow-up radiographs showed complete disappearance of the calcifying mass 6 months after the initial visit to our hospital, without recurrence during a follow-up period of more than 2 years.

**Lessons:**

Acute calcific periarthritis is diagnosed based on history, clinical examination, and imaging findings, which provide evidence for the diagnosis of calcium deposition in the digits even if the lesions have been present for a long time. Watchful observation is an appropriate treatment strategy for acute calcific periarthritis of the digits.

## 1 Introduction

Acute calcium deposits most commonly develop around the shoulder, and are frequently detected on radiographs. However, it is rare for similar deposits to develop in the interphalangeal joints of the digits, including the distal and proximal interphalangeal (PIP) joints of the fingers and the interphalangeal joint of the thumb.^[1–24]^ These calcium deposits have been given various names, but are currently classified into 2 types: acute calcific arthritis and peritendinitis.^[2]^

Acute calcium deposits are referred to as acute calcific periarthritis if they are located in a periarticular region, and as acute calcific peritendinitis if they are present within a tendon.^[2,3]^ When these calcium deposits occur in the digits, they can cause diagnostic confusion, with differential diagnoses including calcifying or ossifying lesions. Acute calcific arthritis and peritendinitis follow a self-limiting disease process, typically showing mild improvement during the first week and resolution within 1 month.^[2,25]^

Acute calcium deposits are reportedly occasionally misdiagnosed, and the patients are treated with antibiotics or unnecessary surgery. We herein describe a rare case of acute calcific periarthritis around the PIP joint of the left fifth finger that took a long time to spontaneously resolve, and review previous reports of similar cases.

## 2 Case report

A 69-year-old woman presented with a 19-month history of pain and an enlarging soft tissue mass in the ulnar aspect of the PIP joint of the fifth finger of the left hand. She was a housewife who performed no particular work or sporting activity. She had a history of minor trauma involving bruising of the finger in a door and was referred to a neighboring clinic 14 months before the visit to our hospital. Plain radiographs taken at the previous clinic had shown no sign of fracture, but instead revealed an abnormal calcifying lesion of the soft tissue of the left fifth finger. She had visited several clinics and hospitals and had been treated with analgesics and splinting for more than 2 months, but the pain in the finger had gradually worsened. Thus, she was referred to our hospital for definitive diagnosis and treatment.

**Figure 1:**
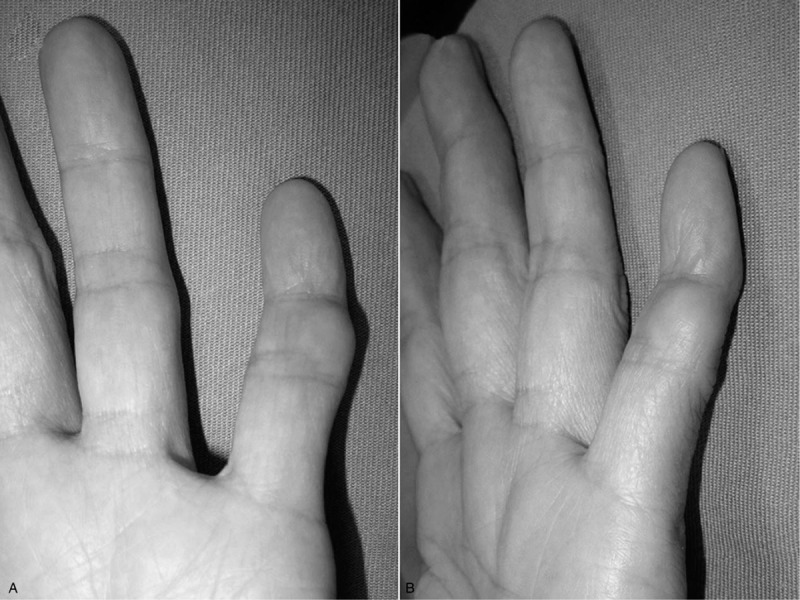
Initial presenting clinical photographs showing a subcutaneous mass around the proximal interphalangeal joint of the left fifth finger. (A) Frontal view. (B) Lateral view.

Physical examination revealed tenderness around the PIP joint of the fifth finger with an apparent subcutaneous tumor, measuring around 1 cm in diameter (Figs. 1A and B). She experienced pain around the PIP joint of the fifth finger when the fist was tightly clenched and/or when the lesion contacted another object. There were no signs of infection or neurovascular disturbances, and no history of previous infection. The range of motion of the affected PIP joint was slightly more restricted than that of the contralateral side, but there was no functional impairment of the finger. Blood chemistry analysis showed no signs of inflammation or other abnormalities.

**Figure 2:**
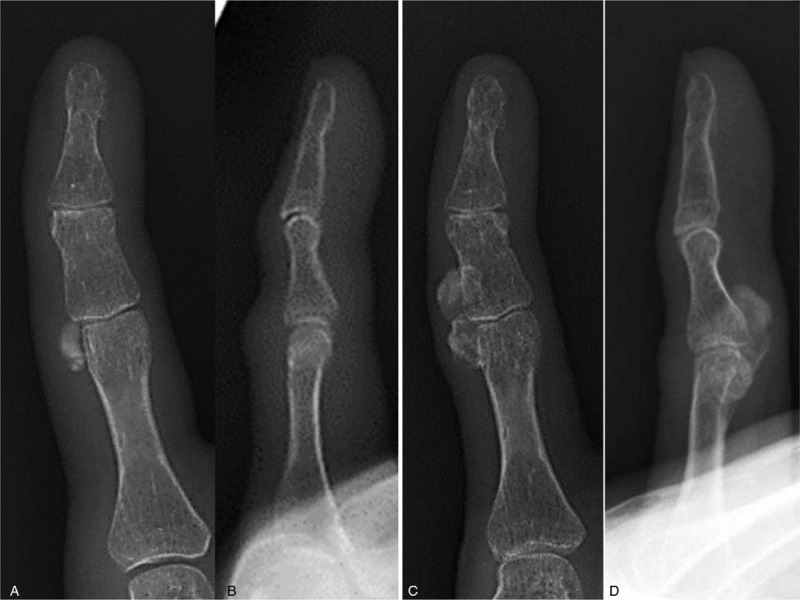
Plain (A) anteroposterior and (B) lateral radiographs taken at a neighboring clinic, showing a subcutaneous calcifying lesion (0.6 cm × 0.2 cm) on the ulnar side of the proximal phalangeal head of the left fifth finger. Initial presenting (C) anteroposterior and (D) lateral radiographs taken at our hospital, showing a 2-humped subcutaneous calcifying lesion (1.3 cm × 0.5 cm) on the ulnar side of the proximal interphalangeal joint of the left fifth finger.

**Figure 3:**
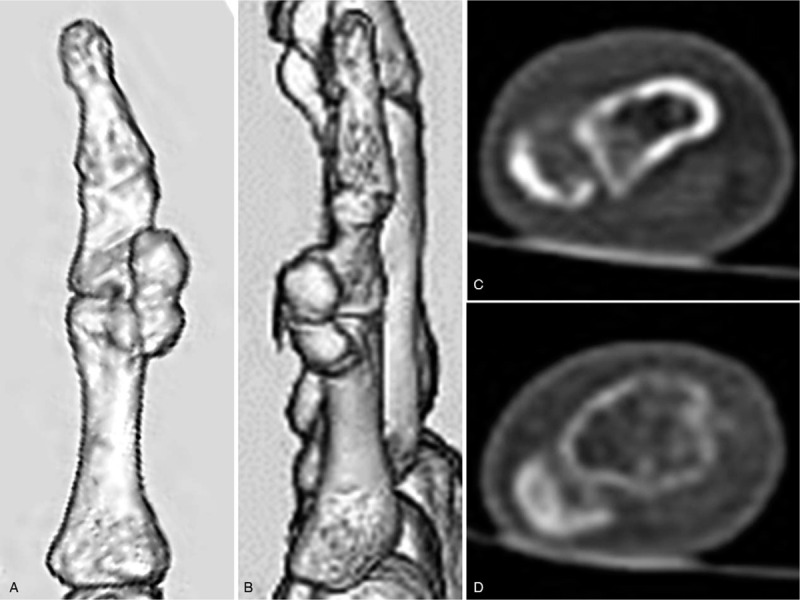
(A) Frontal and (B) lateral 3-dimensional computed tomography views of the left fifth finger, showing subcutaneous bony prominences on the ulnar aspect of the middle and proximal phalanx. Cross-sectional image of the (C) middle and (D) proximal left fifth phalanges, showing a well-defined soft tissue mass overlying the ulnar side of the middle and proximal phalanges with evidence of calcification; the lesion is well separated from the adjacent bone and there is no periosteal reaction or bony destruction.

**Figure 4:**
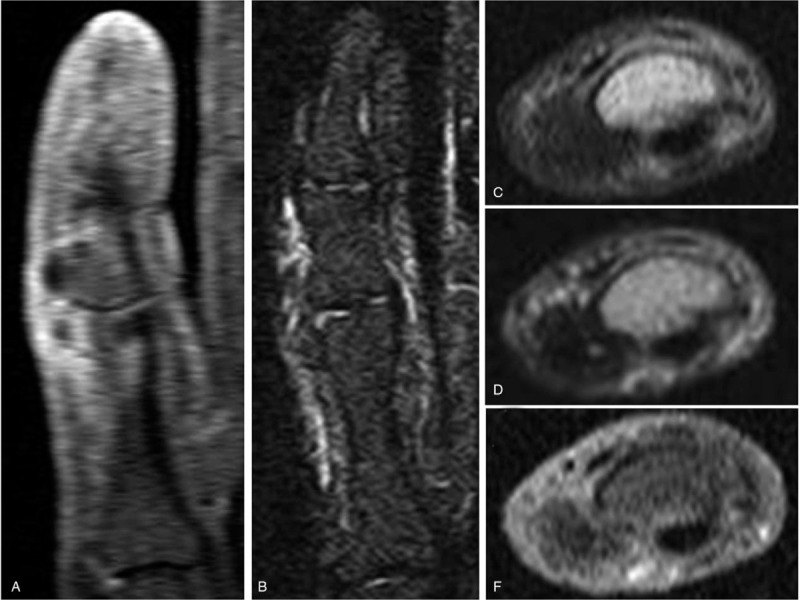
Magnetic resonance imaging of the ulnar side of the proximal and middle fifth phalanges, showing a well-defined humped soft tissue mass with low signal intensity surrounded by a diffuse high signal intensity area on (A) T1-weighted and (B) T2-weighted images. Cross-sectional magnetic resonance imaging of the proximal interphalangeal joint of the left fifth finger, showing that the lesion was well separated from the adjacent bone with no periosteal reaction on (C) T1-weighted, (D) T2-weighted, and (E) contrast-enhanced images.

Plain radiographs of the fifth finger taken 5 months before the initial visit to our hospital revealed a well-defined calcified soft tissue mass overlying the ulnar side of the proximal and middle phalanges that was well separated from the adjacent bone, with no periosteal reaction (Figs. 2A and B). Radiographs taken at the time of presentation at our hospital revealed an enlarged 2-humped calcifying lesion overlying the ulnar side of the PIP joint (Figs. 2C and D). Computed tomography also showed a well-defined and rimmed calcifying soft tissue mass with calcification of the outer margins on the ulnar side of the left fifth finger, without bony destruction (Figs. 3A–D). T1- and T2-weighted magnetic resonance imaging (MRI) showed a well-defined soft tissue mass with low signal intensity overlying the ulnar side of the proximal and middle phalanges (Figs. 4A–D). T1-weighted MRI also showed that the lesion was well separated from the adjacent bone and surrounded by a diffuse high-intensity area, suggesting perilesional soft tissue edema. No periosteal reaction was detected. Moreover, there was no abnormal intensity in the bone marrow observed on either T1- or T2-weighted MRI, suggesting no progression to the bone marrow. Contrast-enhanced MRI showed no enhancement of the soft tissue mass (Fig. 4E). There was no cartilaginous matrix formation. Taken together, these imaging modalities indicated a well-defined subcutaneous calcifying mass with a characteristic peripheral radiopaque ring overlying the ulnar side of the proximal and middle phalanges, suggesting a benign calcifying lesion, namely a calcifying deposit. An excisional biopsy was recommended to achieve a definitive diagnosis, but this was declined by the patient. Thus, no invasive treatments were administered, and she was treated with analgesics and encouraged to massage the affected finger.

**Figure 5:**
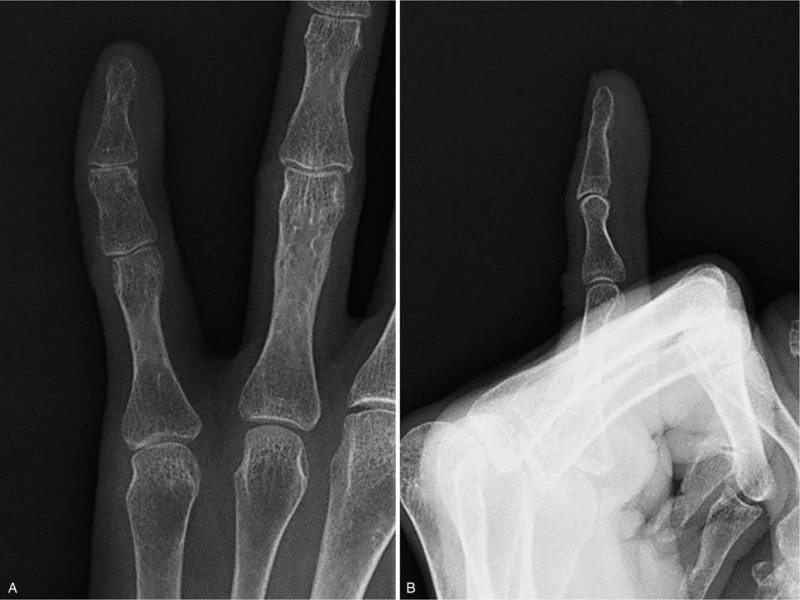
Plain (A) anteroposterior and (B) lateral radiographs taken 6 months after the initial presentation to our hospital, showing complete disappearance of the calcifying lesion around the proximal interphalangeal joint.

The pain in the left fifth finger gradually improved during the following 6 months. In addition, the limited range of motion completely recovered, and follow-up radiographs showed complete resolution of the calcifying mass at 6 months after the initial visit to our hospital (Figs. 5A and B). At the final follow-up conducted 3 years after the initial visit to our hospital, the patient had a full range of motion without recurrence of acute calcific arthritis.

## 3 Discussion

Acute calcific periarthritis in the digits is fairly rare,^[1–24]^ and large case series have only been reported in 3 studies. Sandstrom ^[26]^ reported finger involvement in only 6 of 329 cases (1.6%) of peritendinitis carcarea in 1938, Carroll and Sinton^[27]^ reported finger involvement in 16 of 100 patients (16%) with acute calcareous deposits of the hand and wrist in 1955, and Yelton and Dickey^[28]^ reported finger involvement in 16 of 107 patients (15%) with calcification of the hand and wrist in 1958. Moreover, a review of the English literature related to acute calcific periarthritis reported that acute calcific periarthritis was observed in 20 joints in 15 patients.^[29]^ To our knowledge, acute calcific periarthritis in adults has only been reported in the English literature in 69 digits and 74 phalangeal joints in 61 patients (5 males, 19 females, and 37 patients of unspecified sex) (Table 1 ). Although the sex of 37 patients was not reported, acute calcific periarthritis or peritendinitis is more frequently reported in females than in males, which is consistent with previous reports of acute calcific periarthritis or peritendinitis in the hand and wrist.^[11,29]^ According to previously reported cases, the most frequently affected interphalangeal joint is the PIP joint, which is consistent with our case. However, acute calcific periarthritis with long-term symptoms and a residual mass seems to be uncommon.

**Table 1:**
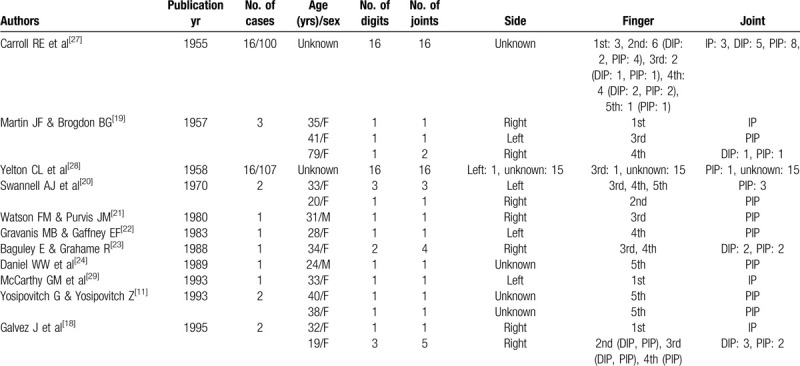
Summary of all known published reports of acute calcific periarthritis or peritendinitis of the fingers.

**Table 1 (Continued):**
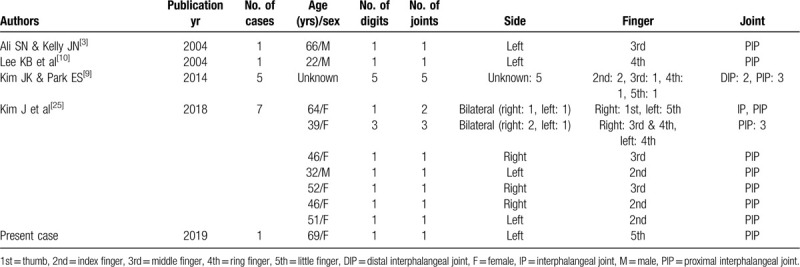
Summary of all known published reports of acute calcific periarthritis or peritendinitis of the fingers.

The clinical presentation of acute calcific periarthritis typically involves rapid onset of monoarticular pain that spontaneously resolves within several weeks. Typical symptoms include swelling, erythema, and/or fever.^[11,27]^ Laboratory inflammatory markers including complete blood count, C-reactive protein, and erythrocyte sedimentation rate are typically normal, with negative cultures.^[2,30]^ Although acute calcific periarthritis resolves spontaneously with or without specific treatment, the condition may be mistaken for other pathological conditions. Because of the rarity of acute calcific periarthritis of the digits, the definitive diagnosis of this condition can be challenging, and it is particularly difficult to attain a definitive diagnosis and perform appropriate treatment when the symptoms and residual mass persist for a long time, as in our case. Although advanced imaging is usually not necessary for the diagnosis of acute calcific periarthritis,^[30]^ computed tomography or MRI are required when the condition persists for a long time. In our case, although the lesion was present for more than a year, computed tomography and MRI provided a diagnosis of calcium deposits in the finger.

The differential diagnoses for acute calcific periarthritis include other benign calcifying or ossifying lesions.^[4–6,9,11,30]^ The other benign calcifying lesions include gout, pseudogout, tumoral calcinosis, or a more concerning infectious etiology (flexor tenosynovitis, septic joint, or osteomyelitis). However, gout typically has associated erosive bony changes, pseudogout presents with a linear chondrocalcinosis, and infection typically does not present as a radiographic calcification.^[6,9,30]^ Benign ossifying lesions include fracture callus, myositis ossificans,^[31–34]^ fibrous reactive periostitis,^[35,36]^ bizarre parosteal osteochondromatous proliferation,^[35,36]^ acquired osteochondroma (Turret exostosis),^[35,36]^ and subungual exostosis.^[35,36]^ Fracture callus and myositis ossificans are associated with a remote history of trauma,^[37]^ and complete spontaneous resolution of these lesions has not been reported. Fibrous reactive periostitis,^[35,36]^ bizarre parosteal osteochondromatous proliferation,^[35,36]^ acquired osteochondroma (Turret exostosis),^[35,36]^ and subungual exostosis^[35,36]^ show similar clinical and radiological features and belong to the same group of reactive lesions of the bone surface^[35,36]^; although these ossifying lesions occasionally resemble acute calcific periarthritis on imaging, they rarely undergo complete resolution.^[35,38]^

In the present case, radiographs and computed tomography revealed a well-defined subcutaneous mass composed of dense, amorphous, homogenous, cloudlike, round, or oval calcific deposits. Additionally, there was calcification of the outer margins on the volar and ulnar sides of the soft tissue of the finger without bony destruction, and the lesions were well separated from the adjacent bone on the ulnar side of the fifth finger. MRI showed that the calcifying lesion had a thin rim of low signal intensity at its boundaries, which were surrounded by diffuse perilesional soft tissue edema on T1- and T2-weighted imaging; there was no abnormal intensity in the bone marrow, suggesting no progression to the bone marrow. All imaging findings suggested a benign calcifying lesion or calcium deposit.^[32,38,39]^

As the clinical course of acute calcific periarthritis is self-limiting and typically resolves over the course of 1 month, the condition is effectively treated via non-surgical treatment comprising rest, icing, and non-steroidal anti-inflammatory drugs.^[2,30,40]^ Although the exact pathological mechanism of these calcium deposits remains unclear, they are thought to develop due to a mechanical or vascular insult that results in poor tissue oxygenation and metaplasia.^[41]^ Hypoxia in the critical area of the ligament or tendon initiates calcific periarthritis or peritendinitis and fibrocartilaginous metaplasia, which results in the formation of calcium deposits. Acute calcifying periarthritis reportedly involves precalcific, formative, resorptive, and healing phases,^[41]^ which are distinguishable on radiographs. The metaplastic tissue undergoes calcific deposition and eventual resorption and healing.^[30]^ Severe pain is typically associated with the resorptive phase of the disease process.^[42]^ A more rapid resolution of pain is reportedly achieved via injection with local anesthetic with or without steroids.^[1,9,11,27]^ However, it is unclear whether the quicker symptomatic resolution is due to mechanical destruction of the calcific mass by the needle resulting in a greater surface area for spontaneous resorption or due to the actions of the medication itself. Some authors have performed surgical intervention for persistent lesions and/or recurrent lesions in the hand and wrist.^[30]^ However, there were no recurrent lesions reported in a series of 17 patients with acute calcific periarthritis around the hand and digits during 12 months of follow-up.[9] In the present case, the pain and calcifying lesion persisted for more than a year, and the complete resolution of acute calcific periarthritis took a long time. Although it is unclear why the pain and the lesion persisted for more than a year in our case, this persistence might have been due to the large size of the calcifying lesion.

Although the risk of recurrence of acute calcific periarthritis is still unknown, watchful observation is generally recommended for calcium deposits.^[33]^ In our case, the acute calcifying periarthritis eventually completely resolved without residual pain and/or complications. Moreover, lesion recurrence has not been observed. Our case suggests that watchful observation might be the appropriate treatment for acute calcifying periarthritis, even if the lesions are present for a long time.

## 4 Conclusion

We have reported a case of complete resolution of acute calcifying periarthritis of the fifth finger. Acute calcific periarthritis of the digits is sometimes misdiagnosed due to its rarity and its broad list of differential diagnoses. However, acute calcifying periarthritis of the digits can be diagnosed on the basis of history, clinical examination, and imaging findings, even if the lesions are present for a long time. Watchful observation is an appropriate treatment strategy for acute calcifying periarthritis.

## Acknowledgments

The authors thank Kelly Zammit, BVSc, from Edanz Group (www.edanzediting.com/ac), for editing a draft of this manuscript.

## Author contributions

**Investigation:** Yuji Tomori.

**Writing – original draft:** Yuji Tomori.

**Writing – review & editing:** Yuji Tomori, Mitsuhiko Nanno, Shinro Takai.
